# HW/SW Platform for Measurement and Evaluation of Ultrasonic Underwater Communications

**DOI:** 10.3390/s22176514

**Published:** 2022-08-29

**Authors:** Unai Fernández-Plazaola, Jesús López-Fernández, Eduardo Martos-Naya, José F. Paris, Francisco Javier Cañete

**Affiliations:** Communications and Signal Processing Lab, Telecommunication Research Institute (TELMA), ETS Ingeniería de Telecomunicación, Universidad de Málaga, 29010 Málaga, Spain

**Keywords:** underwater acoustic communications, ultrasonic frequencies, fading channels, broadband channel measurements, channel estimation, sounding signals, OFDM, MIMO, filter bank, Doppler spread

## Abstract

The purpose of this work is to present a flexible system that supports the study of wideband underwater acoustic communications (UAC). It has been developed both to measure channels and to test transmission techniques under realistic conditions in the ultrasonic band. This platform consists of a hardware (HW) part that includes multiple hydrophones, projectors, analog front-ends, acquisition boards, and computers, and a software (SW) part for the generation, reception, and management of acoustic sounding signals and noise. UAC channels are among the most hostile ones and exhibit an important attenuation and distortion, essentially due to both multipath propagation, which results in a very long channel impulse response, and time-varying behavior, which produces a notable Doppler spread. To cope with this challenging medium, sophisticated transmission techniques must be employed. In this sense, adequate signal processing algorithms have been designed aiming not only at the analysis and characterization of underwater communication channels but also at the evaluation of diverse modulation, detection, and coding schemes, from Orthogonal Frequency Division Multiplexing (OFDM) to single-carrier digital modulations with a single-input multiple-output (SIMO) configuration that takes advantage of diversity techniques. Wideband sounding signals, to be injected into the sea from the transmitter side, are created with patterns that allow multiple tests on a batch. With offline processing of the captured data at the receiver side, different trials can be carried out in a very flexible manner. The different aspects of the platform are described in detail: the HW equipment used, the SW interface to control acquisition boards, and the signal processing algorithms to estimate the UAC channel response. The platform allows the analysis and design of new proposals for underwater communications systems that improve the performance of the current ones.

## 1. Introduction

This paper is focused on underwater acoustic communications (UAC) for shallow water applications and deals with a measurement system designed for UAC channels in the ultrasonic range. In this section, we give context to the work, especially from an experimental perspective.

UAC channels impose relevant restrictions like strong attenuation, mainly due to water absorption; severe time-dispersion, because of multipath propagation; and also, frequency-dispersion, due to the Doppler broadening caused by the medium and terminals motion [1,2,3]. All these factors impede the use of high data-rate communication systems [4,5], because the high delay spread and the low speed of sound put UAC channels around the limit, even crossing it, of being overspread [6,7,8,9]. Hence, the applications are mostly oriented to low-rate communications, like a collection of data from sensor networks, environmental monitoring, surveillance, etc. [10].

Several works have reported measurements of sea trials trying to characterize both the UAC channel response and the received additive noise. The authors of [11,12], provide estimations of the multipath channel impulse response obtained by means of single-carrier sounding signals, with a direct sequence spread spectrum (DSSS) scheme and binary phase-shift keying (PSK) modulation at a carrier frequency of 40 kHz. In [3], a wide set of trials are described, with signals located in bandwidths up to 32 kHz, to analyze the channel behavior with emphasis on its doubly selective nature, in time and frequency. Less attention has been devoted to noise measurements, but we mention [1] that gives a general view of the expected power spectral density (PSD) and [13] that presents some trials to study UAC noise in the audio frequency band in shallow water scenarios and provides estimations of the noise PSD as well.

There are interesting papers that deal with communication system trials for lower frequencies and for ultrasonic bands. Among the firsts, it is worth mentioning the pioneering work in [14], which describes experimental tests in a lake in deep waters, the authors employed a frequency-hopping scheme with PSK signals to reach data transmissions of about 5–6 kb/s. The more recent work in [15] analyzes the experimental behavior of a communication system working at audio frequencies in a trial at the sea with a water depth of 100 m approximately, using a SIMO configuration and comparing diverse techniques based on an OFDM system for low data rates. Likewise, multiple-input multiple-output (MIMO) systems have been deployed in shallow water scenarios, for narrow-band signals in the audible frequencies, and their performance is discussed in [16,17,18]. Other notable works that show measurements of systems in similar scenarios and operating at the ultrasonic band, but in low frequencies around 30 kHz, can be found in [19,20].

There are few efforts trying to develop systems for higher bit-rate applications, like e.g., video transmission, although of modest quality [21]. Some interesting ideas of how to transmit video signal through UAC channels are provided in [22], proposing a selective frequency mapping of the video components according to their importance, with simulation tests over modeled channels for a band from 4 to 30 kHz. Likely, the more ambitious experimental UAC trial for video transmission of wide bandwidth and at high ultrasonic frequencies (between 60 and 100 kHz) is reported in [23]. Although the link is established at deep waters (at 100 m depth), with more favorable channel conditions, and off-line simulations were performed to apply the receiver algorithms over the recorded data, which included adaptive decision-feedback equalizers whose complexity is not described. There exist also works presenting measurement trials for other applications like in [24] for underwater acoustic localization, by using DSSS signals at 15 kHz, or for military applications ([25] and references therein), but in this latter case the technical details are not usually unveiled. All this literature supports the increasing interest in the development of new systems that improves the current technology for UAC applications.

The main contribution of this paper is to describe the details of a platform that has been designed for UAC measurements in our research group. It is based on the signal processing of sounding signals that are injected into the water from a transmitter and are registered at a remote receiver. Both terminals are autonomous and contain specific hardware (HW) equipment that is controlled by ad-hoc software applications. The presented set-up allows carrying out trials in underwater scenarios over a wide band of ultrasonic frequencies, up to approximately 200 kHz. Nevertheless, there is a trade-off between bandwidth (and, hence, data rate) and signal-to-noise ratio (SNR), because the higher the frequency the higher the UAC channel attenuation due to the water absorption [1]. For that reason, we limit our measurements up to 130 kHz. The presented platform has been successfully used both in narrow-band UAC trials, for channel characterization [26], and in broad-band UAC trials, with a twofold aim: channel characterization [9] and communication systems assessment based on OFDM [27]. To the best of the authors’ knowledge, no research group has published any description of this type of platform in such detail. This paper will facilitate the work of those researchers who want to create their own measurement setups. In addition, a signal processing algorithm for estimating UAC channels based on filter banks is presented and its better performance is justified as compared with other classical ones based on a correlator receiver.

We summarize here the main distinctive facts of our system that makes it innovative:

1. High operating frequencies in the ultrasonic band, 32–128 kHz.
2. Very broad bandwidth of 96 kHz, two octaves.
3. A robust and rather automated system that allows registering long signal records.

The first one is a challenge, because the UAC channel conditions at such frequencies are more hostile, the transducers operations are less favorable and it is more difficult to reach long distances. The second one represents a trade-off. On one hand, the SNR is less homogeneous in the whole frequency band and receiver algorithms must face more difficulties to reach good performance. On the other, we can exploit the diversity in frequency, e.g., by means of coded OFDM [27], and employ apparently simpler schemes, with lower spectral efficiencies, but effective and reliable. The third one is an advantage to studying the time-varying nature of UAC channels with higher resolution [9] and testing broadband communication systems that deal with it.

The paper is organized as follows. After this introduction, we will describe the UAC scenarios that are targeted in the trials. In the third section, the equipment employed to carry out the measurements is explained whereas, in the fourth one, it is the software used for the control and management of the involved signals that are shown. The fifth section deals with the algorithms designed for signal processing of the data obtained from the trials and, in particular, for synchronization and channel estimation. Finally, some conclusions are given in the last section.

## 2. UAC Scenarios

The measurement and emulation platform presented in this paper is designed to investigate certain scenarios in the field of UAC, especially within the 3-octave ultrasonic band from 32 kHz to 128 kHz. This section describes in detail these communication scenarios and their associated UAC channels.

**Figure 1:**
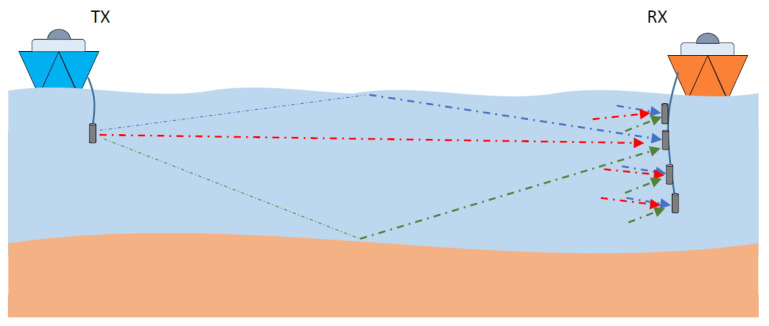
1 × 4 SIMO UAC horizontal channel.

At first, our platform is designed for the measurement/emulation of both horizontal and vertical UAC channels. The *horizontal UAC channel* is characteristic of shallow waters in littoral zones. As shown in Figure 1, possible transmission paths include direct line-of-sight (LoS) (red), surface reflection (blue) and bottom reflection (green). There is an essential difference between the two reflections mentioned. Reflection at the surface usually occurs with a reflection coefficient whose modulus is close to one due to the large difference between the characteristic impedance of seawater and air, this implies that little energy is lost in such reflection. On the other hand, reflection at the bottom depends very much on the geological nature of it. If the bottom is sandy, a significant part of the incident energy is usually absorbed, while if the bottom is rocky, little energy is lost for similar reasons to what occurs at the seawater-air interface. The full configuration for the UAC horizontal channel is also shown in Figure 1. The platform is able to measure/emulate a 1 × 4 single-input multiple-output (SIMO) UAC channel, i.e., transmitting through one projector and receiving through up to four hydrophones. The number of receiver hydrophones can be variable from one to four, including UAC single-input single-output (SISO) channel measurement.

**Figure 2:**
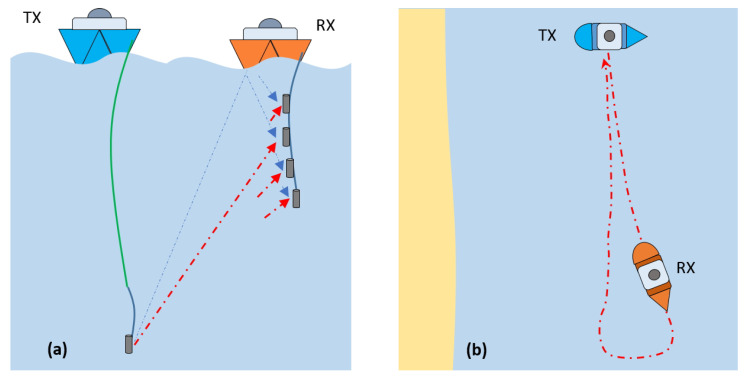
Diagrams for UAC measurement scenarios: (**a**) 1 × 4 SIMO vertical channel; (**b**) dynamic channel.

The *vertical UAC channel* is shown in Figure 2a. In this case, the paths followed by the acoustic waves are predominantly orthogonal to the bottom plane and the effect of reflection on the seabed is non-existent or almost negligible. In general, the vertical UAC channel is much less hostile for communication than the horizontal one. The main reason is that multipath components other than LoS usually reach the receiver in a smaller number, with lower energy and with less temporal dispersion. As shown in Figure 2a, measuring vertical channels at the receiver requires an extension cable, which in some scenarios can be large (the green cable). In addition to horizontal and vertical UAC channels, the platform is capable of measuring other types of geometric configurations that are a mixture of the two.

Secondly, the platform is capable of measuring both quasi-static and dynamic UAC channels. *Quasi-static channels* are those in which both the transmitter and receiver do not explicitly move relative to each other, and channel variations are due to oscillations of both around a given equilibrium position, as well as variations and movements of the physical environment (waves, currents, tides, marine life, etc.). However, quasi-static UAC channels in the ultrasonic band exhibit strong temporal variations due to the low speed of sound propagation in seawater (≈1500 m/s). *Dynamic channels* are those in which there is explicit relative motion between the transmitter and receiver. Figure 2b shows a dynamic channel with a looped path, which is one of those commonly used in our measurement platform. Dynamic UAC channels with modest TX-RX relative velocities, around or greater than 1 sea knot, easily become overspread channels in the ultrasonic band; that is, the channel coherence time is equal to or less than the effective duration of the impulse response.

**Figure 3:**
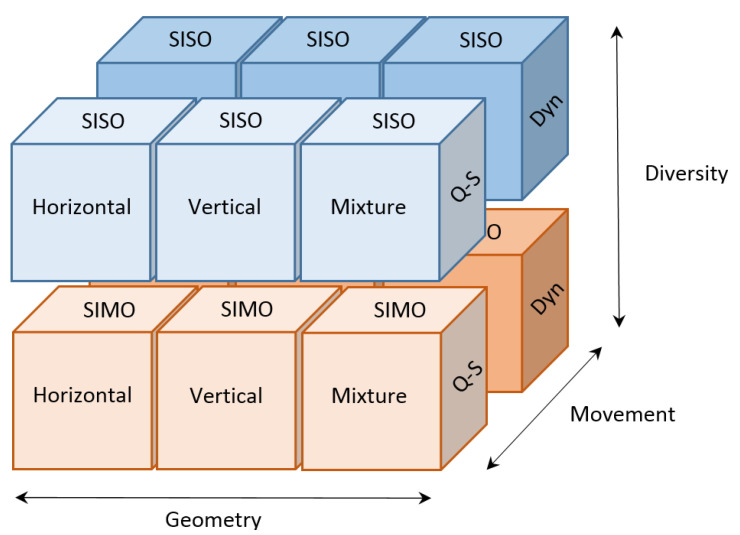
Summary of the UAC scenarios supported by the measurement platform according to geometry (horizontal, vertical, and mixture), movement (quasi-static and dynamic) and diversity in reception (SISO and SIMO).

Finally, Figure 3 summarizes twelve basic scenarios covered by the measurement/emulation platform for three different dimensions: scenario geometry, relative motion between communication nodes, and use or not of diversity in reception.

## 3. HW of the Measurement/Emulation System

**Figure 4:**
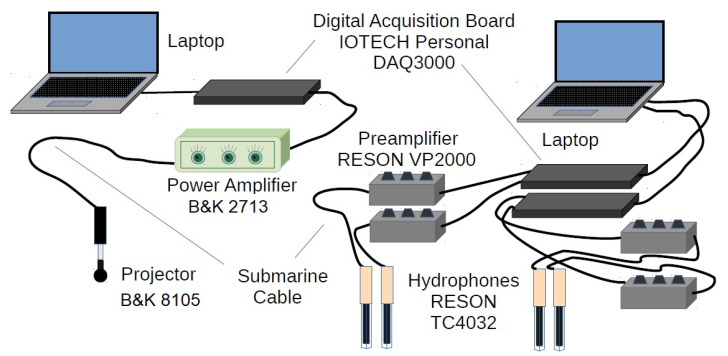
Schematic of the platform HW.

The HW of the measurement/emulation platform is shown schematically in the Figure 4. On the transmitter side, the test signal is generated by the platform SW (explained in the next section) and sent to the IOTECH Personal DAQ 3000 board that performs the digital to analog (D/A) conversion. This board is capable of generating a transmitted signal at a sampling frequency of 1 MHz and with a resolution of 16 bits. Before being injected into the projector, the signal is boosted by the classic Bruel&Kjaer 2713 power amplifier. The Bruel&Kjaer 8105 projector is quite omnidirectional and its frequency response is of the resonant type, reaching its resonance peak at around 100 kHz. Although the non-flat response of the projector must be taken into account in estimating the acoustic response of the channel. The projector response, in the band of interest, is mainly high pass and, since both the underwater channel response and the noise power spectral density are low pass [1], the overall result is that similar values of SNR are obtained throughout all frequencies. On the receiver side, the platform is capable of supporting up to a maximum of 4 RESON TC4032 hydrophones. Each pair of hydrophones use an IOTECH Personal DAQ 3000 board that has the analog to digital (A/D) conversion function. This is because the effective sampling frequency for each channel is divided by the number of channels used. Hence, in order to limit the maximum frequency of the acquired signals as little as possible, we decide to use one card for the transmitter and two for the receiver. This way the transmitter card can use a sampling frequency of up to 1 MHz and the receiver cards of up to 500 kHz per channel, which is enough for the maximum working frequency we are targeting of 128 kHz (that implies a minimum sampling frequency of 256 kHz). In contrast to the projector, the RESON TC4032 hydrophone has a fairly flat frequency response and needs a preamplifier like the RESON VP2000 before the A/D conversion. This hydrophone has a spatial response quite omnidirectional as well.

**Figure 5:**
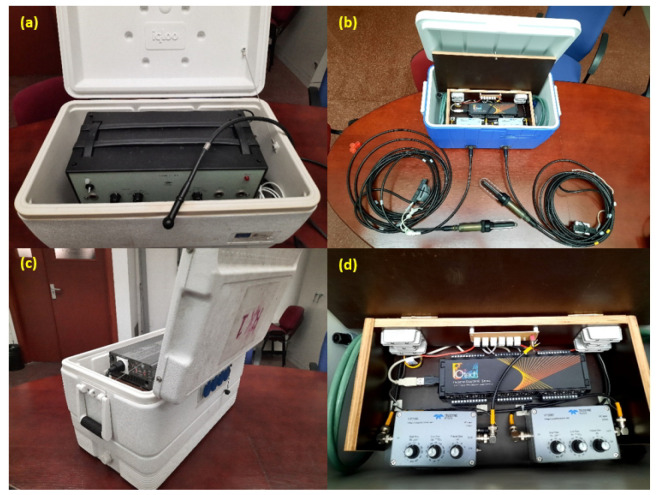
Images of the HW platform implementation: (**a**) transmitter unit, (**b**) receiver unit for 2 hydrophones, (**c**) power inverter unit and (**d**) top view of the receiver unit.

In Figure 5, we summarize the physical implementation of the HW platform. Figure 5a shows the transmitter unit, which is packaged in a watertight box protecting the power amplifier, and also the projector. Figure 5b presents the receiver unit for a pair of hydrophones, inside its watertight box, consisting of two preamplifiers (one for each hydrophone) and a shared acquisition board; the platform has two such units to support 4 hydrophones. Figure 5c shows the inverter power supply unit, capable of providing both 12 VDC and 220 VAC; the 220 VAC output is essential to feed the transmitter unit power amplifier. This power unit is connected to the transmitter and receiver units through watertight cables and connectors. Finally, Figure 5d presents a more detailed picture of the receiver unit, where the amplification gain knob and the cutoff frequency knobs of the preamplifier can be seen.

## 4. SW of the Measurement/Emulation System

The SW of the system has been developed to manage and control the realization of measurement campaigns. Laptop screens are not easy to see at sea outside on the deck of the ships, especially on sunny days, which makes SW operation more difficult than under normal conditions. Therefore, the interface is designed with large buttons so that the measurements can be configured with a minimum of user actions. The SW of the system consists of two applications. The first one is the transmitter app, which controls the acquisition board that generates the input signal for the amplifier connected to the projector. The second one is the receiver app, which controls the two acquisition cards in charge of acquiring the signals received by the 4 hydrophones. The configuration of a measurement requires specifying the particular conditions: distance between the transmitter and receiver boats, amplifier gains, separation between the hydrophones, swell conditions, depth, etc. The SW has been developed so that for each measurement configuration a different set of transmitted signals can be specified. To optimize the process, all these signals are transmitted sequentially, without user intervention. However, they are separated by silences, i.e., intervals in which nothing is transmitted. These silences allow the receiver app, by means of basic signal processing, to split the received signals and save each one in separate files. This greatly facilitates the measurement organization and its subsequent analysis. Despite that, everything received is recorded and stored in files, i.e., no received signal sample is discarded, because the silence periods serve later as noise measurements.

The SW of the measurement system has been developed in Microsoft Visual C++ using the Applications Program Interface (API) called DAQX. It is an API provided by the manufacturer IOTECH for its cards and valid for the Personal DAQ3000 [28]. Although the manufacturer offers other SWs to control the card, the API is the one that allows better control of the card operation and easier embedding in custom applications. As mentioned in the previous section, the cards work with sampling frequencies up to 1 MHz at the transmitter and up to 500 kHz per channel/hydrophone at the receiver.

### 4.1. Transmitter Application

The operation of the transmitter SW is relatively simple since it is only necessary to specify which set of signals, from the previously configured ones, are to be transmitted. The specific conditions of the measurement are not configured in the transmitter but in the receiver. By means of text files, a button is configured for each set of signals to be transmitted. The duration of each signal and the silence time among them are also configured in these files. Samples of each signal are provided by Matlab files.

Two transmission modes are considered: fixed duration and indefinite duration. In the fixed duration mode, the set of configured signals is transmitted, each with its predefined duration, ending when the last signal is sent. This is the mode used to measure quasi-static channels described in Section 2. In the indefinite duration mode, the same signal is transmitted all the time until the user cancels the transmission. The indefinite mode is interesting, for instance, to measure dynamic channels (described in Section 2) to study the effect of movement when the distance between the boat changes, and we want to analyze the effect on the same signal. To inform the user about the transmission status, the application indicates which signal is currently being transmitted from the selected set of signals or if it is in a silence period.

The most significant details in the transmitter app implementation are those related to the DAQ3000 card programming. Using the API, the card is configured in a mode that allows transmitting any signal (with the parameter *DdomDynamicWave* of the *daqDacSetOutputMode* function) and it can work either in a fixed duration mode or in an indefinite duration mode. (In the fixed duration mode, the card is configured with the *DdwmNShot* parameter of the *daqDacWaveSetMode* function, in which the number of samples to be transmitted must be specified. In the indefinite duration mode, the *DdwmInfinite* parameter is specified.) Likewise, the card internal clock is selected as the reference for the D/A conversion. The signal samples to be generated are loaded into a buffer (by using the *daqDacWaveSetBuffer* function). Once the card is configured to transmit, i.e., when the card is armed, the signal generation starts and it ends when the specified number of samples is reached in the fixed duration mode or when the card is disarmed in the indefinite duration mode if the user presses the end button. Silences between signals are simply non-transmitting states, i.e., time intervals with the card disarmed.

### 4.2. Receiver Application

**Figure 6:**
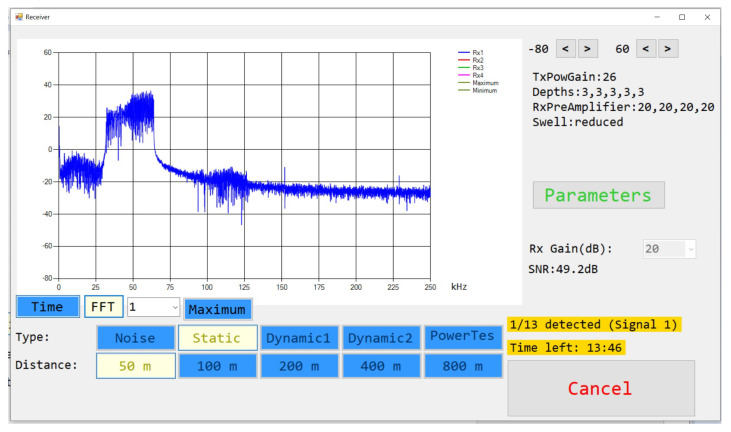
Appearance of Receiver application interface.

Figure 6 presents the appearance of the receiver application interface. The buttons are fully configurable by means of text files, as well as the combination of them that defines a type of measurement. In the example shown in the figure, the first row has been configured so that determines the set of signals to be used and the second one the distance between the boats. The parameters button allows us to specify other characteristics of the measurement to be performed: power used in the transmitter, the gain of the receiver preamplifiers, depth at which the hydrophones are, swell… Another control allows us to specify the gain that the card applies to the acquired signals. During the course of measurement and with a configurable refresh rate, a graph of the received signal is displayed, either in time or in frequency using the Fast Fourier Transform (FFT). As the signals are received, the application performs a basic signal processing to estimate the received SNR, to activate a signal saturation alarm, as well as to separate the set of received signals and save each one in a different file. The saturation alarm allows us to check if the gains applied either to the transmitter amplifier, the receiver preamplifiers, or the acquisition card, are not adequate for the distance between the transmitter and the receiver. In such case, the user can readjust gains and restart the measurement.

We describe the details of the receiver application implementation that are related to the DAQ3000 card programming, acquisition optimization, and the measurement-saving loop. The card is configured so that once the measurement is started it does not end until the user presses the end button which disarms the card. This way, no received signal sample is lost. The card allows defining different gains so that the analog input signal dynamic range fits the card input range, which is selectable between the larger limits, +/−10 V, and the smaller one, +/−0.1 V. The selection is done by setting an amplitude gain of 1 to 100, respectively. The SW allows the user to define which gain to apply according to the needs. The board is configured so that the reading buffer, which is a circular buffer, is managed by itself (by selecting both the *DatmDriverBuf* and *DatmCycleOn* mode). The card generates an overload event if it has to write in the buffer by overwriting samples that have not yet been read. Depending on the laptop performance, an overload event may be more or less likely. The application is designed so that, in the event of an overload, the acquisition continues and is not canceled. Nevertheless, a procedure is implemented to try to avoid overload, as described later.

**Figure 7:**
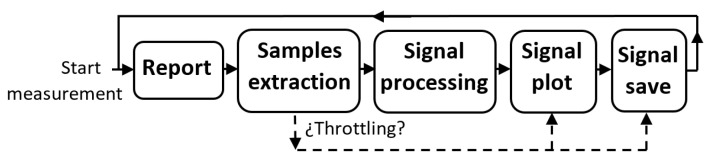
Measurement Loop diagram.

The number of samples to request in each call is made configurable, although the usual value we choose for this record length is 10,000, which corresponds to a time interval of 20 ms for the sampling frequency of 500 kHz. In order to check how far we are from an overload, we calculate the waiting time of the reading samples function (samples are read from the card by the *daqAdcTransferBufData* function using the *DabtmWait* mode, whereby the function does not terminate until all requested samples are available). The percentage of this time with respect to 20ms gives a metric that allows us to anticipate possible overloads. We call *throttling* to a situation in which a high probability of overload is expected. Once the measurement is started, the application enters into a loop and performs the actions shown in the diagram of Figure 7 in each iteration. First, information on the capture status is displayed on the interface (signal being expected, saturation detection, estimated SNR, etc.). Second, the specified number of samples of the record length is obtained from the card and it is evaluated whether to get in throttling mode or not. Then, a basic signal processing is applied to the samples and, later, a short-time windowed signal is plotted on the screen, either in the time or frequency domain. Finally, the signal segment is recorded on disk.

When the application enters in throttling mode, and to avoid overload, some actions are stopped. The frequency at which the received signal is painted on the screen is configurable, but if the application is in throttling mode, painting is stopped. On the other hand, in normal conditions the samples are recorded on disk as they are obtained, but the file is only closed when a configurable time elapses, the default value is 1 s. The file closing action is when the operating system consumes the most time. There is a trade-off between choosing a very long period of file closing that reduces the total CPU time but increases the risk of sample loss in case of a fatal error (e.g., the laptop runs out of battery). In the latter case, all samples in memory since the last time the file was closed would be lost. Into the throttling mode, although this involves more risk, the closing of files is postponed to reduce the probability of overload.

There are two kinds of tasks involved in the signal processing of the measurement loop. A first group, carried out each iteration, includes a filtering in the band of interest, from 32 to 128 kHz, and a local count of clipped samples from the maximum and minimum values registered in each segment, among others. A second group is performed after a configurable number of iterations, which is 5 by default and corresponds to an extended interval of 100 ms. They comprise the detection of the sounding signal beginning; the signal end; a saturation/clipping event. To detect the signal beginning and end, the power in the 100 ms interval is estimated. Since the set of transmitted signals are separated by silences, it is possible to detect significant power variations when changing from silence to signal or vice versa and a differential algorithm compares the power level of successive extended intervals to decide, by means of heuristic thresholds, when a signal is starting or ending. To validate a signal start detection, it is verified that the power level remains high over some time, to avoid that spurious in the received signal could lead to false start detections. The signal detection does not influence the signal recording, which is always done from the first sample, but allows to change the processing phase towards the detection of the signal end that does determine the current file closing (and automatically a new file is open to keep on saving the following samples). Regarding the detection of a saturation event, in each extended interval, the number of clipped samples is estimated by accumulating the local maxima/minima of each signal segment and if it exceeds a configurable threshold, a saturation alarm is generated. In a similar fashion to the transmitter app, the receiver app shows the evolution of the detected signal, so that the user can follow the transmission status of the selected set of sounding signals.

## 5. Description of the Signal Processing

In this section, we describe the technical details of the algorithms designed for channel estimation, or communication tests, from the sounding signals. This estimation procedure is an end in itself when trying to characterize the channel response from measurements and is a means when we want to demodulate the received signals to assess the performance of a given communication system.

### 5.1. Procedure for Channel Estimation Based on Multicarrier Signals

We denote the time-invariant impulse and frequency responses of the channel as h(τ) and H(f) respectively while the sampled counterparts will be denoted as $h\left[n\right]=h\left(nT_{s}\right)$ for n=0…N−1 and $H\left[k\right]=H\left(\frac{k}{NT_{s}}\right)$ where $T_{s}$ is the sampling frequency and N is such that $NT_{s}$ is longer than the duration of h(τ). In the general case of a time-variant channel, the impulse and frequency responses will be written as h(t,τ) and H(t,f) respectively and their discrete-time versions $h\left[m,n\right]=h\left(mNT_{s},nT_{s}\right)$ and $H\left[m,k\right]=H\left(mNT_{s},\frac{k}{NT_{s}}\right)$.

One of the most widespread channel sounding systems is the correlative sounder [3,7,18] which consists in transmitting repetitively a probe signal p[n], performing correlation at the receiver and storing successive channel snapshots. Common probe signals are linear frequency modulated (LFM) chirps and pseudo noise (PN) sequences due to their favorable autocorrelation properties, i.e., Φ[n]≡p[n]⊛p[−n]≈δ[n] where ⊛ stands for the *N*-point circular convolution. The signal processing carried out by the correlative sounder can be described as follows.

Let $\tilde{p}\left[n\right]$ denote the periodic sounding signal and let *N* and *L* be the length of p[n] and the number of successive transmitted copies of p[n], respectively. Hence $\tilde{p}\left[n\right]=\sum_{m=0}^{L-1}p\left[n-mN\right]$. Assuming that the impulse response of the channel h[n] is static (this restriction will be relaxed later), the received signal $\tilde{y}\left[n\right]=\tilde{p}\left[n\right]*h\left[n\right]$ will also be periodic. Since the convolution of a periodic signal $\tilde{p}\left[n\right]$ (with a period of *N* samples) with a non-periodic signal h[n], with $N_{h}\leq N$ samples and $n=0\ldots N_{h}$, can be expressed as the concatenation of the *N*-point circular convolution of p[n] and h[n], it follows that $\tilde{y}\left[n\right]=\sum_{m=0}^{L-1}y\left[n-mN\right]$ with y[n]=p[n]⊛h[n]. Next, the output of the correlation receiver can be expressed as $\tilde{s}\left[n\right]=\tilde{y}\left[n\right]*p\left[N-n\right]$, where an *N*-point delay has been included so that p[N−n] is defined in the range n=0…N−1. Following the same reasoning as before, we can write $\tilde{s}\left[n\right]=\sum_{m=0}^{L-1}s\left[n-mN\right]$ where s[n]=y[n]⊛p[N−n] and, hence s[n]=p[n]⊛h[n]⊛p[N−n]≈δ[n−N]⊛h[n]=δ[n]⊛h[n]=h[n] and therefore $s\left[n\right]=\hat{h}\left[n\right]$ where $\hat{h}\left[n\right]$ is the estimated impulse response. The output of the correlator receiver can be expressed in a more convenient way as

(1) $$\tilde{s}\left[n\right]=\sum_{m=0}^{L-1}\hat{h}\left[n-mN\right]$$

with

(2) $$\hat{h}\left[n\right]=y\left[n\right]⊛p\left[N-n\right].$$

The output in (1) is composed of a concatenation of successive channel estimations $\hat{h}\left[n\right]$ each of which is computed as indicated in (2). This particular way of describing the correlator system directly leads to an alternative way of implementation using the FFT and inverse FFT (IFFT). Concretely, each period of the received signal in (2) can be obtained as

(3) $$\hat{h}\left[n\right]={\mathrm{IFFT}}_{N}\left\{{\mathrm{FFT}}_{N}\left\{y\left[n\right]\right\}\cdot {\mathrm{FFT}}_{N}\left\{p\left[N-n\right]\right\}\right\}$$

**Figure 8:**
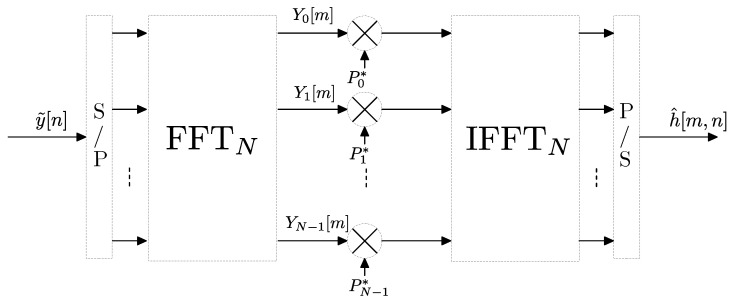
Block diagram of correlator receiver implemented via FFT. P/S stands for parallel to serial conversion and S/P for serial to parallel.

The procedure is shown in the block diagram of Figure 8 where $Y_{k}={\mathrm{FFT}}_{N}\left\{y\left[n\right]\right\}$ and $P_{k}^{*}={\mathrm{FFT}}_{N}\left\{p\left[N-n\right]\right\}$.

**Figure 9:**
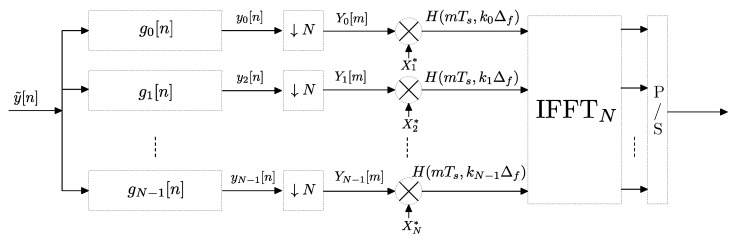
Block diagram of the channel estimation procedure using a bank of filters and a unit amplitude sequence at transmitter.

An alternative to the correlator receiver approach described above is the use of a multitone/multicarrier probe signal at the transmitter and a bank of filters at the receiver, which is the technique we have adopted in our measurement campaigns. In the following derivation we will show that our method is a generalization of the correlator receiver and may offer remarkable advantages. Let $\tilde{x}\left[n\right]=\sum_{k\in K}X_{k}\cdot e^{j\frac{2\pi }{N}kn}$ be a discrete-time complex sounding signal composed of a sum of (at most *N*) tones or carriers equally spaced in frequency where K is a set of positive integers smaller than *N* and where $X_{k}$ is the complex amplitude of the *k*-th carrier. As stated in [29] the peak-to-average power ratio (PAPR) of the sounding signal can be minimized if $X_{k}$ is a *Zadoff-Chu* sequence, i.e., $X_{k}=e^{-j\phi_{k}}$ with $\phi_{k}=\pi qk^{2}$ where *q* is a constant related to the length of the sequence. Assuming a static h[n], the received signal can be written as $\tilde{y}\left[n\right]=\sum_{k\in K}H\left[k\right]\cdot X_{k}\cdot e^{j\frac{2\pi }{N}kn}$. The frequency response H[k] of the channel is estimated using a bank of *N* filters centered at the discrete frequencies of the sounding carriers $\frac{k}{N}$, k=0…N−1. The impulse response of each filter is given by $g_{k}\left[n\right]=g\left[n\right]\cdot e^{j\frac{2\pi }{N}kn}$, n=0…M−1 where g[n] is a low pass filter with cutoff frequency $\frac{1}{2N}$ and length *M* samples, being M≥N. In ideal conditions of flat frequency response of the low pass filter g[n] the output of each filter will be given by $Y_{k}\left[n\right]=H\left[k\right]\cdot X_{k}\cdot e^{j\frac{2\pi }{N}kn}$ i.e., a complex exponential with constant amplitude $H\left[k\right]\cdot X_{k}$. Taking a sample of $Y_{k}\left[n\right]$ at instant n=N results in $Y_{k}=H\left[k\right]\cdot X_{k}$ and the estimation of the frequency response at index *k* is obtained by $\hat{H}\left[k\right]=\frac{Y_{k}}{X_{k}}=Y_{k}\cdot X_{k}^{*}$. Finally, an *N*-point IFFT is applied to obtain $\hat{h}\left[n\right]$. The procedure is shown in the block diagram of Figure 9.

**Figure 10:**
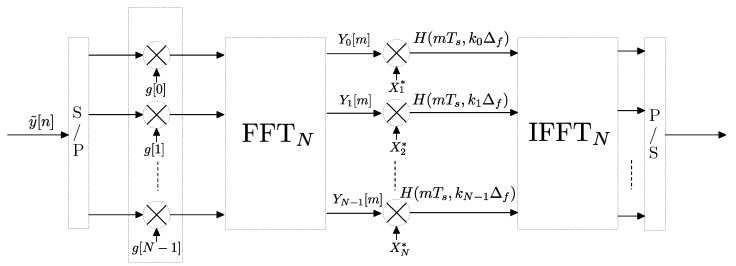
Block diagram of the channel estimation procedure using a multicarrier sounding signal, of unit amplitude and a bank of filters implemented via FFT.

For the simple case of M=N, the bank of filters followed by decimation depicted in Figure 9 can be implemented using the FFT [30] as shown in Figure 10, where the received signal $\tilde{y}\left[n\right]$ is windowed by g[n]. In a more general case, if M≥N, such implementation is still valid by subdividing the *M*-point-windowed input sequence into blocks of *N* points and then stacking and adding these blocks before performing the *N*-point FFT, which is an optimized procedure based on the linear character of the FFT (see the details in [30]).

The comparison of Figure 8 and Figure 10 reveals that both systems are identical except for the windowing. If we consider the received signal in Figure 8 to be windowed by a rectangular window w[n], n=0…N−1, we can conclude that the correlator sounder approach is a particular case of the multicarrier/bank of filters procedure. The equivalent filters of the correlator receiver have an impulse response $c_{k}\left[n\right]=w\left[n\right]e^{j\frac{2\pi }{N}kn}$, for n=0…N−1 and k=0…N−1 which leads to a *sinc*-shaped frequency response. See that the length of each filter is *N* samples.

In order to estimate the acoustic response of the channel it is necessary to compensate for the non-flat responses of both the transmitter’s projector and the receiver’s preamplifier. We compensate both in the frequency domain, before applying the IFFT, with the responses provided by the manufacturer or the ones obtained with a calibration procedure of the equipment setup.

**Figure 11:**
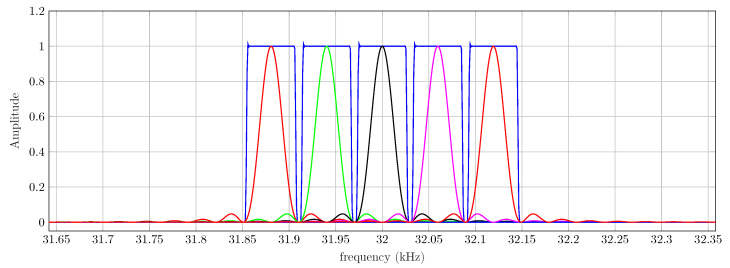
Frequency response of 5 consecutive filters of the bank of filters (blue) and the correlator receiver (other colors).

One of the main advantages of the multicarrier/bank of filters method stems from the fact that the filters $g_{k}\left[n\right]$ can be designed with an arbitrarily close-to-ideal response since there is no restriction in their length *M*. The only drawbacks of using a large *M* are a computational burden increase and an initial delay in the decimation of the output of the filter due to the longer transient of the filters. Both are minor issues since we are dealing with an offline computation. In Figure 11, we have superimposed the frequency response of a set of consecutive filters corresponding to the correlator receiver, $c_{k}\left[n\right]$ (in several colors), and the bank of filters receiver $g_{k}\left[n\right]$ (in blue). In this latter case, the filters have been designed by Hanning-windowing the ideal impulse response and M≫N.

**Figure 12:**
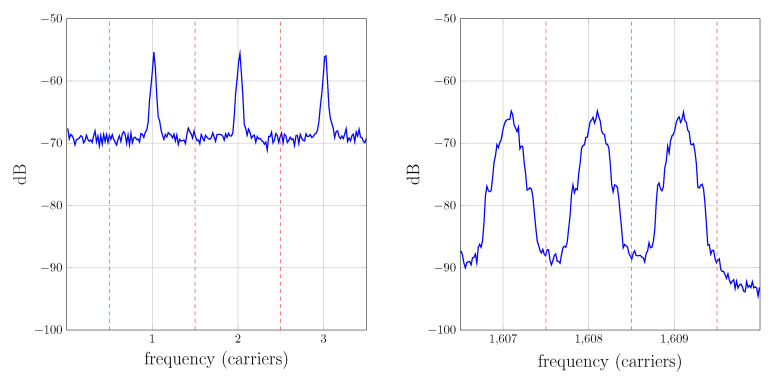
Received carriers spectra before resampling for low index carriers (**left**) and high index carriers (**right**). The vertical dotted lines indicate the filter band boundaries.

If the channel is time-invariant, the received carriers will remain located in their original frequencies and both systems would perform identically. However, in a real situation for UAC scenarios, the time variation of the channel response will lead to a Doppler shift and Doppler spread of the received carriers. In this case, the filters of the correlator receiver may remarkably distort each received tone and moreover, interference from adjacent bands may be significant, because neither the amplitude of the wanted signal would be maximum nor the value of the adjacent signal would be null. This fact is described in Figure 12, where a typical spectrum of received carriers is represented (the information presented in the graphics of this subsection corresponds to a measurement campaign carried out by the authors, whose details are accessible in [9]).

### 5.2. Doppler Effect Reduction by Resampling

**Figure 13:**
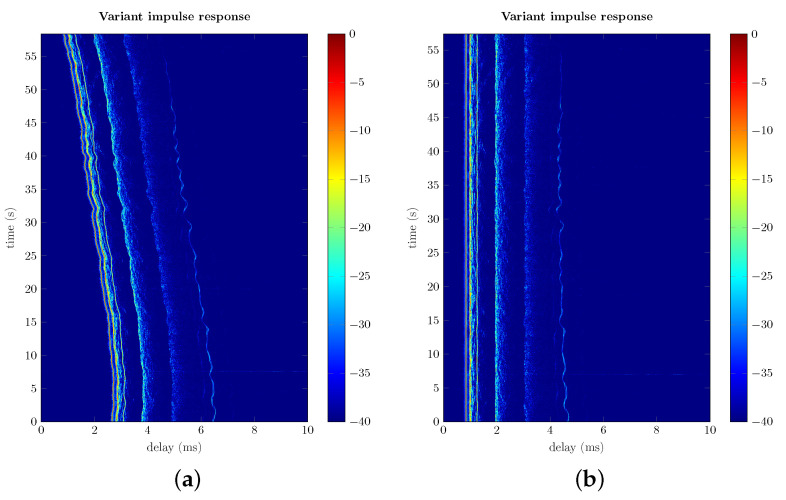
Time-variant impulse response estimation $\hat{h}\left(t,\tau \right)$ of a measured channel before and after resampling. (**a**) Impulse responses before resampling. (**b**) Impulse responses after resampling.

**Figure 14:**
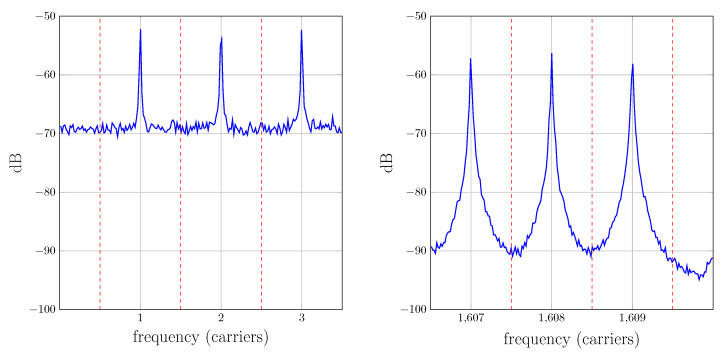
Received carriers spectra after resampling for low index carriers (**left**) and high index carriers (**right**). The vertical dotted lines indicate the band boundaries.

The Doppler spread shown in Figure 12 can be remarkably reduced by preprocessing the received signal using resampling. This topic has been profusely mentioned in the bibliography [2,3,4,7,31,32] but is scarcely detailed. The procedure followed in our measurement campaigns is as follows: first, *L* successive estimations of the impulse response are obtained with the multicarrier/bank of filters method. The fluctuation of the initial delay on each estimation is then used to evaluate the frequency offset produced by the Doppler effect. This offset is compensated by resampling the corresponding block of the received signal. Finally, the impulse response estimation procedure is started over using the resampled received signal, which yields an improved estimation. Figure 13 shows the estimated time-variant impulse response $\hat{h}\left(t,\tau \right)$ of a channel before and after resampling. In that channel, the transmitter was 198 meters from the receiver, the hydrophone and projector were at a depth of 6 meters with respect to the surface and the seabed was at 20 m. The corresponding spectra of the received carriers before and after resampling is depicted in Figure 12 and Figure 14, respectively. Notice that in the latter one, each spectrum is remarkably narrower and closer to the center of its band after resampling.

The fact that the underwater noise spectra strongly decrease with frequency [1] is also noticeable in Figure 12, where the noise floor is significantly higher for the low-frequency tones. Taking into account that these carriers are more confined in their band, we could consider an improved version of the bank of filters receiver of Figure 9 with different cutoff frequency filters, which would further reduce the noise in the estimation.

### 5.3. Enhancing the Measurement System Versatility

The measurement platform can work with any type of signal. For example, in the trials presented in [9], multitone signals were used for channel sounding, with the technique described above. OFDM or single-carrier signals can be employed to analyze and evaluate the performance of any underwater communications system [27]. Since measurement campaigns are generally expensive and time-consuming, it is convenient to choose the transmitted signals so that the same measurements are versatile enough to test different communication schemes. For this purpose, the following strategies have been followed. The signals are generated with non-differential constellations, for example quadriphase shift-keying (QPSK), and modulated with a pseudo-random, but deterministic and known, data sequence that is uncorrelated. If we want to study the behavior of a differential constellation, for example, differential QPSK (DQPSK), this can be achieved by interpreting the received symbols in another way (by differential decoding of the detected symbols). A similar approach can be employed to analyze the effect of using pilots in OFDM for channel estimation with a certain arrangement, more or less dense, by considering the received symbols at a set of carriers as pilots instead of data. Although we select to transmit QPSK constellations, it is also possible to test the behavior of BPSK, with this simple operation

(4) $$\hat{x}=\mathrm{sign}\left(ℜ\left[x_{R}\cdot x_{T}^{*}\right]\right)\cdot x_{T},$$

where the BPSK-equivalent demapped symbol $\hat{x}$ is estimated from an antipodal decision over the scalar product of $x_{R}$ and $x_{T}$, being $x_{R}$ and $x_{T}$ the complex QPSK symbols at receiver and transmitter (which is indeed known), respectively.

Additionally, we adopt the strategies proposed in [33], which allows testing both any type of channel coding and interleaving schemes, and any kind of quadrature amplitude modulation (*M*-QAM). The first is achieved by means of a whitening process and the second by means of a so-called dithering process. In principle, the transmitted signals are generated without applying any type of channel coding. However, a post-processing of the data trials permits us to assess channel coding performance: a whitening binary sequence can be added (with modulo-2 or XOR addition) to the one obtained from the channel encoder under test, so that the resulting sequence is the pseudo-random one used in the measurements. Afterward, at the receiver, such a whitening sequence must be added to the demodulated data before applying de-interleaving and channel decoding. On the other hand, a somehow similar approach is also possible to analyze the behavior of higher order *M*-QAM constellations from the measurement results obtained with a simpler QPSK. For this purpose, a memoryless mapping can be used with a dithering complex sequence that is added at the transmitter after the symbol mapper and is subtracted at the receiver to the samples that feed the desired detector or *M*-QAM symbol demapper (this is a shortened explanation of the more detailed one that can be found in [33]).

Note that the choice of not transmitting modulated signals in measurement campaigns is also possible. Instead, one can use the same approach proposed in [34] (these authors offer a simulator and test channels available for download [35]) and make the convolution of modulated signals with the channel impulse response estimated from measurements, provided that the estimation is sufficiently accurate in time and frequency. In [27], we have presented results using this approach. However, the employ of modulated signals directly in measurement campaigns yields more realistic results. On one hand, the channel estimation is obtained using received signals that are subject to noise and residual distortion (like actual inter-carrier interference). On the other hand, the accuracy obtained in the synchronization algorithms by using periodic sounding signals and resampling, cannot be fully achieved when transmitting modulated signals.

## 6. Conclusions

We have described in this paper a versatile and robust measurement system to perform trials in UAC scenarios. The system comprises two subsystems prepared for on-board operation, one for the transmitter side and the other for the receiver. Both subsystems contain specific purpose HW, among which we highlight projector and hydrophones, analog front-ends, DAQ3000 acquisition boards for A/D and D/A signal conversion, and personal computers. Two SW applications have been developed for the operation, control, and management of both subsystems. Such applications carry out some on-the-fly signal processing tasks to create files where a set of sounding signals are stored after they have been transmitted through the underwater channel. Additionally, offline signal processing algorithms, which are designed to analyze the recorded data, have been explained. These set of algorithms have a two-fold purpose: some are employed to estimate the time and frequency of selective UAC channel response, and others are used to evaluate the performance of digital communication systems under realistic conditions. In particular, the need for signal resampling to compensate for the time expansion and compression it experiences through the UAC channel is addressed. Our system works in the ultrasonic frequency range and manages a wider band than the one used in other reported measurement set-ups, which supports the possibility to reach higher data rate communications. The presented work can pave the way for other research groups interested in carrying out these kinds of trials.

## Data Availability

Not applicable.
